# Space Balls Revisited: Stereological Estimates of Length With Virtual Isotropic Surface Probes

**DOI:** 10.3389/fnana.2018.00049

**Published:** 2018-06-12

**Authors:** Mark J. West

**Affiliations:** Department of Biomedicine, Aarhus University Faculty of Health Sciences, Aarhus, Denmark

**Keywords:** stereology, length, unbiased, isotropic probe, space ball

## Abstract

The space ball probe was fully described in the literature 15 years ago by Mouton et al. (2002). Since then, it has been used in a number of studies in the nervous system that focus on axon, dendrite, and capillary length. The length of structural parameters in tissues reflect functional aspects of the tissues. Here, some of the various applications of this methodology will be presented, along with a review of the salient features of the methodology that has resulted in new wave of quantitative morphological studies of length in the nervous system. The validity of the method is discussed in view of its widespread use along with insights into the problems associated with its application to histological tissue and future techniques for applying space balls.

## Introduction

Estimates of the length of cellular features can provide quantitative information about various biological functions. Examples using space ball probes include; the length of dopaminergic axons, which can be related to the dopaminergic innervation and function of the striatum (Li et al., 2016); the length of serotoninergic (Liu et al., 2011) and cholinergic axons in cerebral cortex which can be related to cortical function (Nikolajsen et al., 2011) and the length of astrocyte processes, which can be related to immune-reactivity (McNeal et al., 2016). These parameters can be used to evaluate brain hemodynamics (Kubíková et al., 2018), tissue oxygenation (Nikolajsen et al., 2015), and tissue repair (Lee et al., 2005; McConnell et al., 2016) and ultimately used to develop therapeutic approaches to brain disorders. Structural parameters of potential interest and for which quantitative studies of length have yet to be carried out include microtubules, involved in intracellular transport, and neuropil threads, an expression of Alzheimer's disease.

## Background

The stereological relationship formula for estimating length density, $\hat{L_{V}}=2•Q_{\text{A}}$

Prior to the introduction of the space ball probe, stereological estimators of length were plagued by the requirement for an isotropic interaction between the area probes and linear features such as axons and capillaries. In this article, the use and application of a virtual isotropic surface probe that readily fulfills this requirement is described.

The formula that relates measurements made on images to length (Smith and Guttman, 1953) is simple and easy to apply (Equation 1). Accordingly, the number of times that a linear structural feature (***Q***) passes through a probe of known area (***A***) is directly related to the length per unit volume (***L***_*V*_) of the structural feature. In spite of the simplicity of the formula, its' derivation is somewhat deep and the derivation and proof are presented in the original Smith-Guttman paper. Briefly, it is based on a three dimensional version of the Buffon needle problem. Unlike the two dimensional Buffon problem, which involves the probability that a randomly thrown needle intersects a line on the floor, the space ball probe is based on the probability that a surface is hit by a line that is randomly oriented in 3D space.

(1) $$\begin{array}{r} \hat{L_{V}}=2•Q_{\text{A}} \end{array}$$

It is important to remember that this formula is applicable only when the linear structural feature has an *isotropic interaction* with the areal probe. This will be the case when the structural feature is truly isotropic. That is, the direction of its linear elements is equal in all three dimensions of space. In this case, the orientation of the 2 dimensional area probe is not important. However, biological structures are seldom if ever isotropic structures. One would prefer to avoid having to make any assumptions about the extent or existence of isotropy in biological tissue in order to avoid potential biases in the estimate that result from anisotropy. Methodological biases of this type lead to estimates that systematically deviate from the true value regardless of the amount of sampling performed.

In order to avoid the problem related to the anisotropy of biological structures a number of techniques have been developed to ensure an isotropic interaction between probe and structural feature. One way to accomplish this would be to section small samples of randomly oriented pieces of tissue and probe these sections with flat 2-D area probes (Nyengaard and Gundersen, 1992; Løkkegaard et al., 2001) or by cutting the tissue in three orthogonal planes (Kubíková et al., 2018). Another would be to use probes with surfaces that were isotropic, such as a sphere (Mouton et al., 2002). In the first case, to fulfill the isotropic interaction requirement, the tissue is randomly oriented. In the latter, the surface of the probe is itself isotropic. Prior to the advent of the space ball probe a number of methods had been developed for estimating length by using combinations of randomness in the orientations of the probe and the structure. For example methods exist in which the tissue is randomly oriented in two dimensions and the probe is oriented randomly in the third dimension (Baddeley et al., 1986), cut in orthogonal planes (Mattfeldt et al., 1985) or in which flat surface probes are randomly oriented in tissue cut arbitrarily (Larsen et al., 1998). Though all of these earlier approaches provide unbiased estimates of length, some are more difficult to implement than others. Area probes with isotropic surfaces (space balls) are generally preferred in view due to the ease with which they can be applied to modern 3D tissue imaging.

## The preferred isotropic spherical probe or space ball

**Figure 1:**
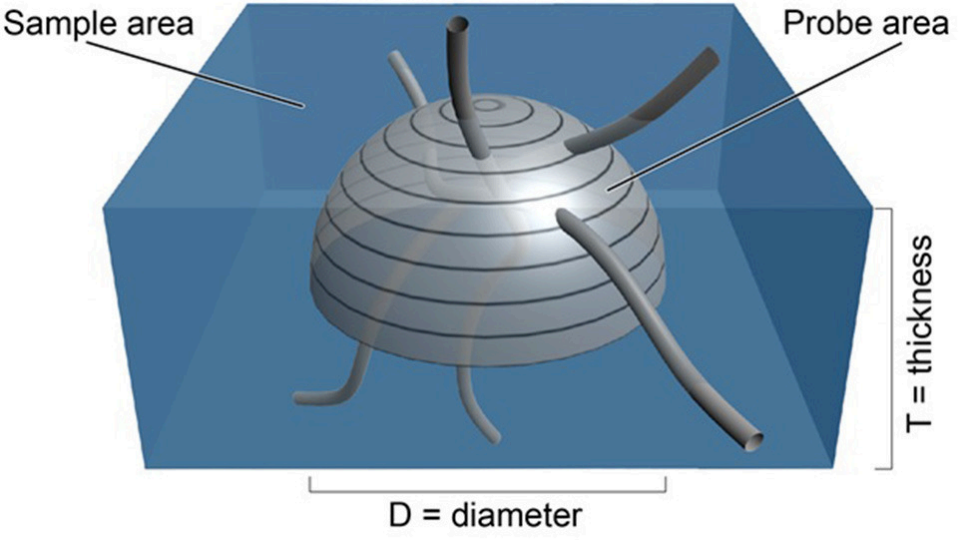
Depiction of an isotropic surface probe shown in light gray in a tissue sample shown in blue. The estimate of the length density, $\hat{L_{V}}$, is directly related to the number of times the linear features pass through the surface of the hemi-space ball probe (see Equation 1). The area of the probe is known from the formula for the surface of a sphere of known diameter, D. The volume of tissue sampled is known from the sample area times the thickness of the section (West, 2012, Figure 5.3, with permission).

The surface of a sphere is an isotropic probe in that all surface orientations are represented on its surface (see appendix in Mouton et al., 2002). In fact each orientation of a surface element on a sphere is represented twice; at the antipodes. As a consequence, the probe need only consist of a hemisphere, since all orientations of the surface are present on the surface of a hemisphere (see Figure 1). By placing a hemisphere, of known area (***A*** = ***2***π ∙ ***r***^2^**,** where ***r*** is the radius of the hemisphere), in a thick section or stack of aligned sections, the area of the probe can be set to any desired value. One would only have to count the number of times a linear feature passed through the surface of the probe to obtain the ***Q*** in Equation (1). Using a hemisphere rather than a sphere also increases the amount of area that the probe can have in a section of a certain thickness.

**Figure 2:**
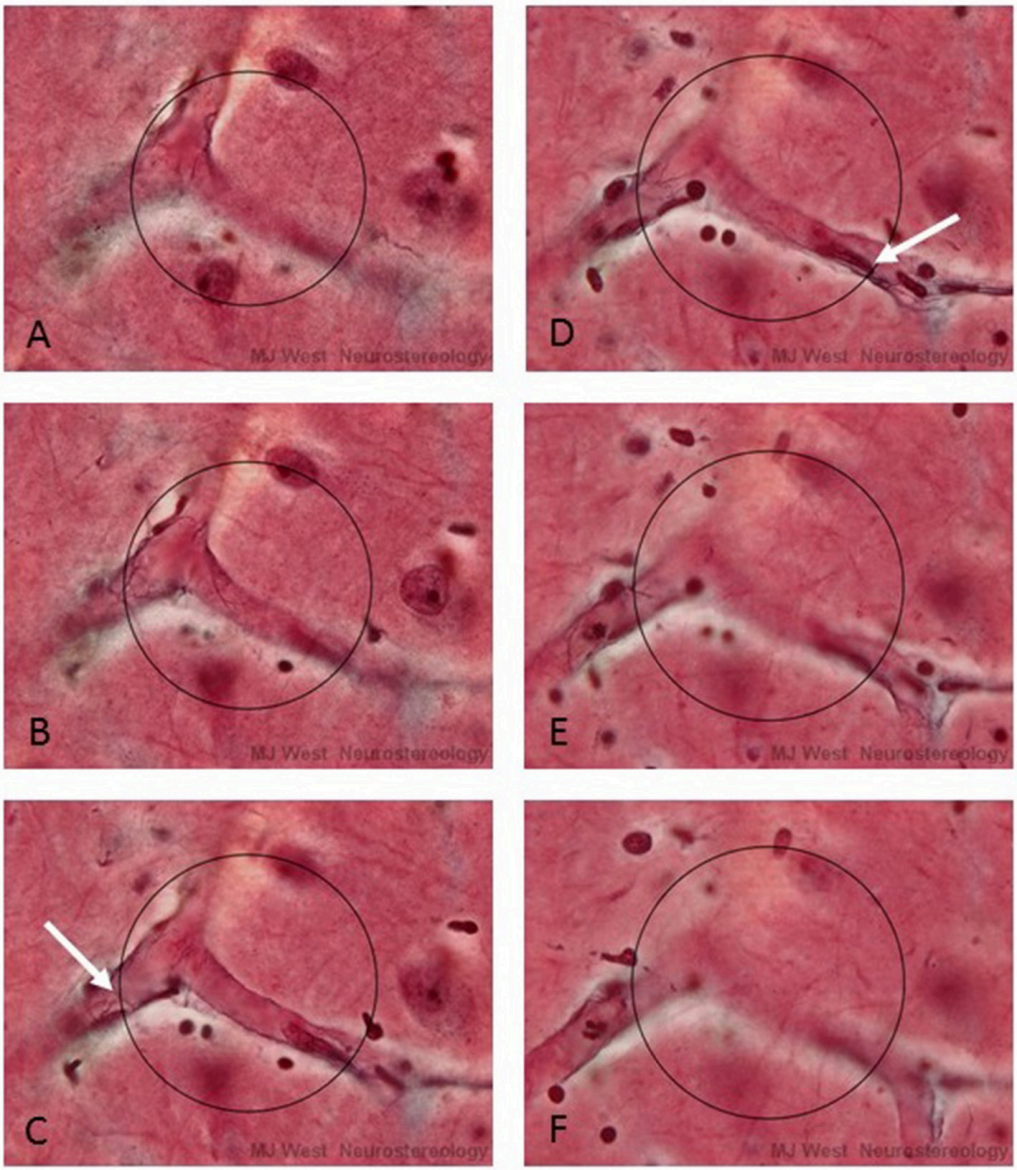
A series of micrographs depicting a virtual hemispheric probe (circles) at six different focal levels **(A–F)** of a thick histological section stained for capillaries. The white arrows in C and D point to interactions, ***Q***, between capillaries and the surface of the probe. In practice the surface of the hemi-space ball appears as an expanding circle as one focuses through the section.

It is impossible to physically place a 3-D probe into histological material. One can do this in a *virtual* manner, however, by superimposing an image of the surface of the probe (which will appear as a circle of a certain size), at particular depths of a thick section or stack of images. By focusing up and down through the tissue or stack, one would see a series of concentric circles of various diameters that represented the surface of the hemispheric probe. At each level, one would determine whether a linear structural feature of interest intercepted the circle that represented the surface of the probe (See Figure 2). This approach is used in commercially available computerized microscope systems that superimpose the surface of the probe, seen as a series of concentric circles, at appropriate focal depths to create a virtual hemispheric probe.

Using thick section light microscopy or stack based confocal and multiphoton microscopy; the choice of using probes with isotropic surfaces is attractive because one can orient the tissue of interest in any convenient plane. This is particularly valuable when one prefers to examine sections that are cut at a particular orientation. This will likely reduce the effort needed to define the borders of the region of interest. The latter is required if one is to make estimates of total length. This important aspect of modern stereology, total amounts, is discussed further below.

## Counting interactions between linear structures and virtual spherical probes

There are a two important points to be made about counting intercepts of the linear feature with the virtual isotropic hemispheric probe. The first has to do with the fact that a capillary, or any biological linear structural feature, for that matter, is not a true line. Linear biological features have a certain diameter, true lines do not. Linear biological structures are often tubes or cylinders. The Smith-Guttman formula applies only to true lines. The diameter of biological linear features can create problems when determining whether or not a linear structural feature interacts with the surface of the hemispheric surface probe. This is particularly so when the diameter of the hemi-space ball is of the same magnitude as the diameter of the linear feature. If one counts intercepts when any part of the structural feature touches the surface of the probe, one will over count the intercepts.

There is a “correction” for the “over counting” that will occur when one uses the touch counting rule. Briefly, one divides the touch intercept counts (***Q***) by 1 plus the ratio of the square of the diameter of the structural feature, ***d***, to the square of the diameter of the space ball probe, ***D***[***1*** + ***(d***^2^***/D***^2^***)***]. From this relationship between the diameters of the probe and structural features, it can be shown that if one used a probe that has 10 times the diameter of the structural feature, there can be expected to be a bias of about 1% in the touch counts. A bias of this magnitude can be considered to be unimportant for all practical purposes. Still, the correction is an approximation of the magnitude of the bias and estimates based on touch counts are not truly unbiased.

**Figure 3:**
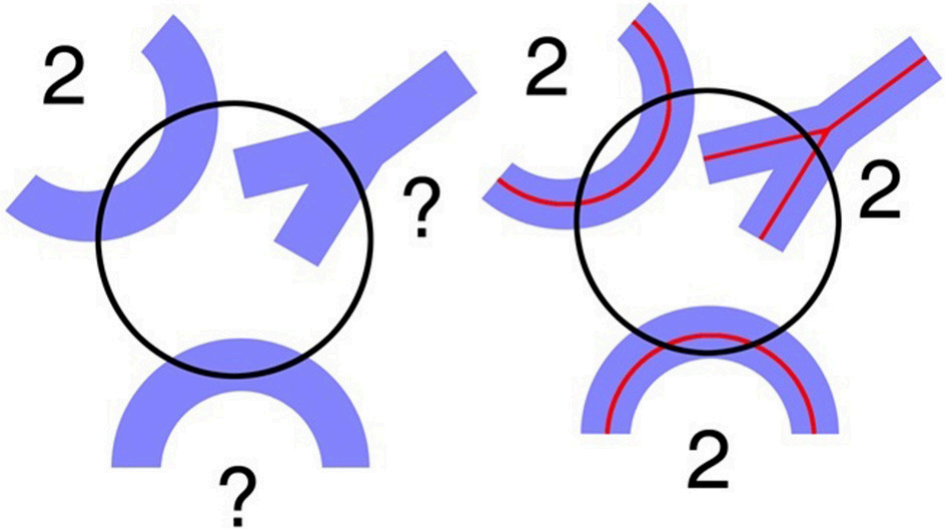
**(Left)** A circle depicting the virtual space ball probe at a specific depth where the linear morphological feature appears in focus. Problems arise when trying to determine how many interactions occur between the probe and the linear morphological features shown in blue. **(Right)** Using the spline counting rule, the number of probe interactions is clearer. There are two interactions for each blue structure (West, 2012, Figure 5.4, with permission).

The second point is that it may be possible to avoid having to correct for “touch” counts. This approach involves defining a central line or spline in the center of a cross section of the linear feature (Figure 3 right). In this case one can avoid having to make corrections for touches. One counts intersections with a one dimensional (true line) spline running along the center of the structural feature. In the case of capillaries, one can readily define the position of a spline midway between the top and bottom of the capillary, i.e., half way through the focal depth of the capillary. The half way point will be readily apparent, in that the capillary wall will be in sharp focus when the endothelial cells are viewed on end at the midpoint a shown in Figure 2. This approach may be attractive when it is not possible to obtain a section or stack thickness that can accommodate a hemi-space ball probe that is five to 10 times the diameter of the structural feature. When this is the case, the estimate will be unbiased for all practical purposes. In the case of very thin fibers, such as cholinergic fibers which are less than a micron in diameter, the ***d/D*** ratio is so small in thick histological sections that one can ignore any bias related to over counting using the touch rule.

## Two ways to make estimates of total length, *L_*TOT*_*

There are two ways to estimate total length. One is to multiply the length density, ***L***_*V*_, by the volume of the region of interest, the reference volume, ***V***_*REF*_. The latter can be readily estimated with point counting techniques.

(2) $$\hat{L}=L_{V}•V_{REF}$$

The other way is to estimate ***L***_*TOT*_ by estimating the length of a structure in a known fraction of the volume of interest and multiplying this by the reciprocal of the fraction of the structure sampled (Mouton et al., 2002). Using the fractionator sampling principle, ***L***_*TOT*_ would be calculated in the following manner,

(3) $$\begin{array}{l} \hat{L}=2\;•\;\Sigma Q\;•\;(1/ssf)\;•\;(v/a)\\ \;=2\;•\;\Sigma Q\;•\;(1/ssf)\;•\;([A_{\text{step}}\;•\;t]/a)\\ \;=2\;•\;\Sigma Q\;•\;(1/ssf)\;•\;([A_{\text{step}}\;•\;t]/(2\pi \;•r^{2})) \end{array}$$

where ***v*** is equal to the volume of tissue sampled by the probe, which is equal to the area associated with the movement from one sampling position to the next, ***A***_*step*_, times the thickness of the section, ***t*** (see blue area in Figure 1). The surface area of the probe, a hemisphere in this case, is equal to ***a***, which is equal to 2π times the radius of the hemisphere, squared ***r***^2^.

To date, over 100 peer reviewed research papers have used the space ball probe (Web of Science). The applications involve a wide range of structural features, ranging from peripheral nerve innervation to central axon terminations, capillary innervation of brain tissue, and gliosis. In a recent study of gliosis (McNeal et al., 2016) the authors evaluated different methods for evaluating cerebral injury the authors concluded; “Only modern stereological techniques (i.e., optical fractionator and space balls) and virtual process thickness measurements demonstrated significant changes in astrocyte number, process length, or proximal process thickness in cases with brain injury relative to controls.”

Since the introduction of space ball probes, there are few scientific papers that have used other unbiased stereological techniques for estimating unbiased length. One example is the exemplary use of the orthogonal triplet probe in the exhaustive work of Kubíková et al. (2018). A recent alternative proposal involve the use the automatic tracking software for following linear features within 3D preparations. Accordingly, 3D reconstructions of the digitized features can be used to estimate length. This approach may ultimately prove to be useful when high contrast imaging and segmentation routines become more available. At present they require significant amounts of operator intervention and monitoring. Automated segmentation of histological features is not yet able to replace the human visual systems ability to rapidly segment structural features in microscopic preparations that vary in background and intensity. This inability, combined with the fact that only slightly more than 100 interactions between probe and structural feature, the “***Q***” in $\hat{L_{V}}=2•Q_{\text{A}}$, are needed to make useful estimates in one individual (West, 2013) render these approaches impractical at this time.

As pointed out in previous publications (Dorph-Petersen et al., 2001; Nikolajsen et al., 2015), tissue shrinkage is a major problem when analyzing length parameters in conventional light microscopic preparations. This because the absolute or true length of linear features, unlike object number, is sensitive to the volumetric distortions in the tissue The increasing use of *in vivo* 3D imaging of biological tissue can be expected to eliminate these problems. For example, a comparison of estimates of length density, using space ball probes in neocortical tissue of DBA mice that had been fixed for light microscopy, 2,857 mm/mm^3^, (Nikolajsen et al., 2015) and in similar mice of the same age, viewed *in vivo* with 2 photon microscopy, (720 mm/mm^3)^ (Gutiérrez-Jiménez et al., 2018) indicate that the length density estimates in the histological material were about four times those for the *in vivo* estimates. This comparison, although limited to only part of the cortex in the *in vivo* material, indicates that the length density of capillaries in light microscopic preparations, increases roughly in proportion to the reduction in volume of the processed tissue. This may not be the case with histological histological material prepared in a manner different from that used in the Nikolajsen study. Interpreting and comparing density data from substantially (and potentially different) shrunken materials across studies should only be done with great care if at all!

## Validation of the methodology

The validation of the space ball method for estimating length is dependent on the validity of the proof of the formula for the relationship between the length of the structural feature and the number of intercepts that occur per unit area of a surface probe, $\hat{L_{V}}=2•Q_{\text{A}}$

That is, the estimate of the length per unit volume, $\hat{L_{V}}$, is equal to 2 times the number of intercepts per unit area ***Q***_*A*_ of probe, with the caveat that the interaction between surface of the areal probe and the linear feature must isotropic. In that this relationship has been proven mathematically (see appendix in Mouton et al., 2002), the relationship itself cannot be validated as such. It is either true or it is not. If proper sampling is used, i.e., the surface of the probes have equal orientations in 3D space and all parts of the region of interest have equal probabilities of being sampled, then the estimate will be unbiased. That is, the estimate will approach the true value of the length without limit, as the amount of sampling is increased (West, 2012).

By definition, the surface of a sphere is isotropic. That is, there are small areas on the surface of a sphere that have all orientations in 3D space (see appendix in Mouton et al., 2002), The use of spherical probes, ensures an isotropic interaction between probe and feature. This will also be so for linear features that are isotropic and anisotropic, so that one does not have to assume anything about the orientation of the feature of interest or the direction along which one sections the material.

The estimation procedure will be assumption free with regard to an isotropic interaction between probe and feature.

If one uses random (unbiased) sampling and isotropic probes, any differences in the results of repeated estimates made with space balls can be related to the amount of sampling performed i.e., the variance of the estimator, or to some inability to meet the other requirements for making an unbiased estimate, i.e., practical issues. The latter might include errors in the delineation of the region of interest, the inability to generate a true spherical probe within a thick section, non-random sampling, histological artifacts, and lack of corrections for tissue shrinkage. Tissue shrinkage is of major concern, in that the length of a structural parameter, unlike object number, is subject to changes if the tissue shrinks. The use of *in vivo* confocal microscopic images removes some of these concerns.

The issue of reproducibility or validity of stereological data obtained with space ball probes is therefore not based on the mathematical relationship equation and the isotropy of the surface of the space ball, but to one's ability to meet the general requirements for making an unbiased estimate. These issues are not trivial and can affect the validity of a particular set of data, but they are not stereological issues. When evaluating the validity of any stereological data, it is essential that one confirms the unbiasedness of the sampling scheme. That is, the unbiased choice of subjects, the unbiased choice of sections, the unbiased choice of the positions on the sections to be probed, and the choice of an unbiased probe. All must be unbiased for the estimate to be valid.

## Author contributions

The author confirms being the sole contributor of this work and approved it for publication.

### Conflict of interest statement

The author declares that the research was conducted in the absence of any commercial or financial relationships that could be construed as a potential conflict of interest.
